# Position Specific Alternative Splicing and Gene Expression Profiles Along the Tonotopic Axis of Chick Cochlea

**DOI:** 10.3389/fmolb.2021.726976

**Published:** 2021-09-08

**Authors:** Heiyeun Koo, Jae Yeon Hwang, Sungbo Jung, Hyeyoung Park, Jinwoong Bok, Juw Won Park

**Affiliations:** ^1^ Department of Anatomy, Yonsei University College of Medicine, Seoul, South Korea; ^2^ BK21 PLUS Project for Medical Science, Yonsei University College of Medicine, Seoul, South Korea; ^3^ Department of Computer Science and Engineering, University of Louisville, Louisville, KY, United States; ^4^ Department of Otorhinolaryngology, Yonsei University College of Medicine, Seoul, South Korea; ^5^ KY INBRE Bioinformatics Core, University of Louisville, Louisville, KY, United States

**Keywords:** alternative splicing, tonotopy, high-throughput sequencing, gene expression profile, cochlea, basilar papilla

## Abstract

Alternative splicing (AS) refers to the production of multiple mRNA isoforms from a single gene due to alternative selection of exons or splice sites during pre-mRNA splicing. It is a primary mechanism of gene regulation in higher eukaryotes and significantly expands the functional complexity of eukaryotic organisms, contributing to animal development and disease. Recent studies have shown that AS also influences functional diversity by affecting the transcriptomic and proteomic profiles in a position-dependent manner in a single organ. The peripheral hearing organ, the cochlea, is organized to detect sounds at different frequencies depending on its location along the longitudinal axis. This unique functional configuration, the tonotopy, is known to be facilitated by differential gene expression along the cochlear duct. We profiled transcriptome-wide gene expression and AS changes that occur within the different positions of chick cochlea. These analyses revealed distinct gene expression profiles and AS, including a splicing program that is unique to tonotopy. Changes in the expression of splicing factors *PTBP3*, *ESRP1*, and *ESRP2* were demonstrated to contribute to position-specific AS. RNA-binding motif enrichment analysis near alternatively spliced exons provided further insight into the combinatorial regulation of AS at different positions by different RNA-binding proteins. These data, along with gene ontology (GO) analysis, represent a comprehensive analysis of the dynamic regulation of AS at different positions in chick cochlea.

## Introduction

Alternative splicing (AS) refers to the production of multiple mRNA isoforms from a single gene due to alternative selection of exons or splice sites during pre-mRNA splicing. It is a primary mechanism of gene regulation in higher eukaryotes and significantly expands the functional complexity of eukaryotic organisms. In humans, over 90% of multi-exon genes are alternatively spliced (Wang et al., 2008). It is well known that defects in alternative splicing are responsible for numerous diseases and maldevelopment, including cancer (Muchir et al., 2000; Eriksson et al., 2003; Muntoni et al., 2003; Morel et al., 2006; Cooper et al., 2009; Giudice et al., 2014; Baralle and Giudice, 2017; Mazin et al., 2021). In addition, previous studies have shown that AS may influence functional diversity by affecting the transcriptomic and proteomic profiles in a position-dependent manner in a single organ (Navaratnam et al., 1997; Rosenblatt et al., 1997; Baralle and Giudice, 2017).

Auditory sensory organs such as the organ of Corti in mammals and the basilar papilla in avians are responsible for receiving and relaying sound information from the environment to the brain. These sensory organs reside within the elongated tubular structure known as the cochlea. Interestingly, auditory receptor cells, the hair cells, are specialized to detect different frequencies of sounds depending on their location along the cochlear duct. The hair cells located at the basal (proximal) regions of the cochlea are tuned to detect high frequencies of sounds, whereas the hair cells at the apical (distal) cochlear regions are tuned to lower frequencies. This position-dependent frequency discrimination capability of the auditory system is known as the tonotopy. Hair cells display gradually different morphological features along the tonotopic axis, which may be crucial for discriminating various sound frequencies. Interestingly, some of the tonotopic features are conserved between the chicken and mouse. For example, the length of the hair bundle, which are composed of tens of stereocilium arranged in a stair-case pattern, is shorter at the base and gradually longer toward the apex, whereas the width of hair bundle is wider at the base and gradually narrower toward the apex (Son et al., 2015; Moon et al., 2020).

Previous studies suggest that tonotopic organization is closely correlated with differential gene expression along the cochlear duct. For example, expression levels of genes related to the sound transduction apparatus, which includes actins, myosins, and ion channels, are tonotopically graded along the chicken cochlea (Frucht et al., 2011). In addition, genes related to signal pathways such as *DNER*, *BMP7*, and retinoic acid are also differentially expressed and regulate morphological features of hair cells important for discriminating sound frequencies in the developing chicken cochlea (Kowalik and Hudspeth, 2011; Mann et al., 2014; Thiede et al., 2014). Moreover, a recent study shows that several genes associated with hearing loss, including *TMC2*, also exhibit graded gene expression patterns along the tonotopic axis in the mature chicken cochlea (Janesick et al., 2021). These results indicate that differential gene expression along the cochlea is a fundamental feature for establishing the tonotopy in the hearing organ.

**FIGURE 1:**
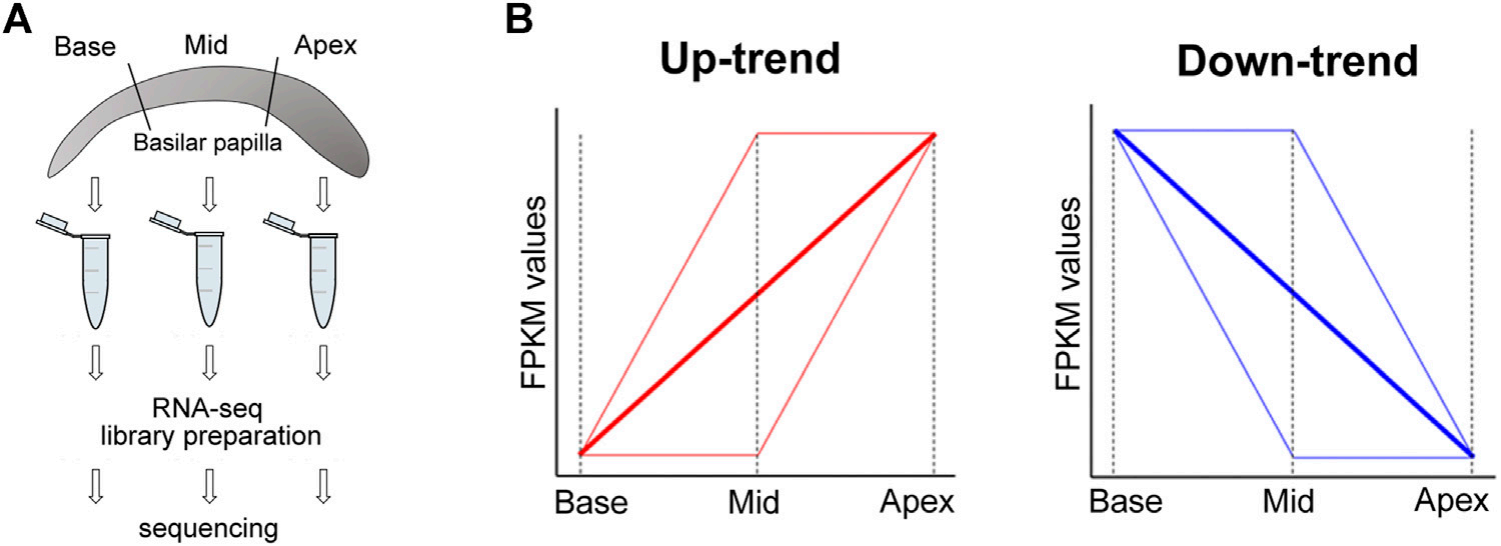
Sample preparation and gene categorization. **(A)** Chick cochlea sample preparation for RNA-seq. Samples are collected and prepared from three regions (base, mid, apex) of the cochlea **(B)** Up-trend genes and Down-trend genes are categorized according to their FPKM value changes along the tonotopic axis (from base to apex).

Unlike differential gene expressions, examples of AS events associated with tonotopy are limited. In the chicken cochlea, detailed analyses have been reported that splice variants of the *KCNMA1* gene encoding Ca_2_^+^-activated K^+^ channels are differentially expressed along the tonotopic axis (Navaratnam et al., 1997; Rosenblatt et al., 1997; Miranda-Rottmann et al., 2010). To systematically identify genes and splicing events whose regulation mechanisms are associated with tonotopy, we conducted transcriptome-wide gene expression profiling and AS analysis using high-throughput sequencing. We used the chicken cochlea because there are ample examples of previously known DEGs along the tonotopic axis and the reported spliced variants of *KCNMA1* can be used as a reference for AS events associated with tonotopy. We extracted mRNAs from three regions (base, middle, and apex) of chick cochlea and performed an RNA-seq experiment (Figure 1A). We identified differentially expressed genes (DEGs) and differentially spliced events from the pairwise comparisons of three regions. The result revealed distinct gene expression profiles and position-specific AS that are unique to tonotopy. We further observed that splicing factors such as *PTBPs*, *ESRP1* and *ESRP2* are differentially expressed along the tonotopic axis. Since these splicing factors are known to regulate the AS events, their position-specific expression may take important roles related to tonotopy by regulating the position-specific AS events.

To show that the results of DEG and AS analyses identified from RNA-seq are accurate, we performed RT-PCR validation on 15 genes and 5 AS events. RT-PCR yielded a perfect validation rate (100%) for both DEG and AS analyses results, confirming that the DEGs and AS events predicted from RNA-seq are true positives.

RNA-binding protein (RBP) motif enrichment analysis near alternatively spliced exons showed motifs of multiple RBPs are enriched near alternatively spliced exons, providing further insight into the combinatorial regulation of AS at different positions by different RBPs. Gene ontology (GO) analysis using genes detected from DEG analysis and AS analysis revealed terms highly related to the developmental process and sound perception.

## Materials and Methods

### Chicken Cochlea Samples and RNA Extraction

Post-hatch day 1 (P1) chickens were euthanized by CO_2_ inhalation followed by decapitation. The head was hemi-sectioned and the cochlea duct was isolated in cold DEPC-treated PBS. The basilar papilla was dissected from the cochlear duct and divided into three pieces: base, middle, and apex. Each piece was collected in a separate Eppendorf tube containing Trizol reagent (Invitrogen, Carlsbad, CA, United States) and quick-frozen to minimize RNA degradation. Total RNA from 12 chickens (24 cochleas) were used for 3 biological replicates for each region (i.e., 4 chickens or 8 cochleas per replicate). RNA was isolated using Trizol reagent according to the manufacturer’s instructions.

### cDNA Generation, and RNA-Sequencing.

We performed an RNA-seq experiment for three sample groups on the base, middle, and apex regions of chick cochlea. Following cochlea dissection and mRNA extraction, the quality and quantity of RNA were examined using Tapestation2100–RNA Screen Tape (Agilent) and a Victor3 Multilavel plate reader (PerkinElmer), respectively. 1μg of total RNA from each sample was reverse transcribed into cDNA and amplified using the Illumina TruSeq RNA Sample Prep Kit v2. For library preparation, mRNA was enriched by oligo-dT to minimize DNA contamination. The PCR products for the cDNA library were purified and selected using the AMPure XP bead (Beckman Coulter). The DNA was quantified using Tapestation2100–D1000 Screen Tape. The cDNA libraries were multiplexed and sequenced on the Illumina Hi-Seq 4,000 platform, and 101-bp paired-end reads were generated. The files from the multiplexed RNA-seq samples were demultiplexed, and fastq files for each sample were generated. Approximately 57–82 million read pairs per replicate were generated. To quantify the transcript abundance, sequencing reads were aligned to the chicken genome (galGal5) and the transcriptome (Ensembl GTF release 91), using the STAR aligner (v2.5.4b) (Dobin et al., 2013), allowing up to 3 mismatches per read.

### Differentially Expressed Gene Analysis and Categorization of Genes Along the Tonotopic Axis

We identified DEGs on base, middle, and apex regions using Cuffdiff (v2.2.1) (Trapnell et al., 2012). We calculated gene expression level as fragments per kilobase of transcript per million mapped reads (FPKM) of each gene based on the length of the gene and the number of reads mapped to the gene. The values of FPKM for genes are included in the Supplementary Table S1. Significant DEGs between two sample groups were identified at FDR <5%, >1.5-fold difference between mean FPKMs, and mean FPKM ≥1.0 in at least one of all three sample groups. These were further categorized depending on the changes of the values of FPKM as Up-trend and Down-trend as below (see Figure 1B for a diagram).

• Up-trend includes genes that show at least a 1.5-fold increase in mean FPKM values with directionality from base to middle to apex, allowing up to 15% variability against the directionality.

$$\frac{\text{FPKM of Apex}}{\text{FPKM of Base}}\geq 1.5\;\text{AND}\frac{\text{FPKM of Middle}}{\text{FPKM of Base}}\geq 0.85\;\text{AND}\frac{\text{FPKM of Apex}}{\text{FPKM of Middle}}\geq 0.85.$$

• Down-trend represents genes that show at least a 1.5-fold decrease in mean FPKM values with directionality from base to middle to apex, allowing up to 15% variability against the directionality.

$$\frac{\text{FPKM of Base}}{\text{FPKM of Apex}}\geq 1.5\;\text{AND}\frac{\text{FPKM of Base}}{\text{FPKM of Middle}}\geq 0.85\;\text{AND}\frac{\text{FPKM of Middle}}{\text{FPKM of Apex}}\geq 0.85.$$

### Alternative Splicing Analysis

We searched differential AS events from the base, middle, and apex regions from RNA-seq data. We identified AS events between two sample groups using rMATS (v3.2.5) (Shen et al., 2012; Park et al., 2013; Shen et al., 2014), which can detect five major types of AS events. Briefly, rMATS uses a modified version of the generalized linear mixed model to detect differential AS from RNA-seq data with replicates. It accounts for exon-specific sequencing coverage in individual samples as well as variation in exon splicing levels among replicates. For each AS event, we used both the reads mapped to the splice junctions and the reads mapped to the exon body as the input for rMATS. In each rMATS run, the first sample group was compared to the second sample group to identify differentially spliced events with an associated change in the percent spliced in (ΔPSI or Δψ) of these events. rMATS was run using the -c 0.0001 parameter to compute *p* values and FDRs of splicing events with a cutoff |Δψ| of >0.01%, then significant splicing events were identified at FDR <5% and |Δψ| ≥ 5% as described previously (Yang et al., 2016).

### Reverse Transcription-PCR for Validation

To validate DEGs and AS events detected from RNA-seq data, we performed reverse transcription (RT)-PCR using extracted total RNA as described previously (Warzecha et al., 2010). Total RNA from chicken P1 cochlear base, middle, apex were isolated using Trizol reagent (Invitrogen, Carlsbad, CA, United States). Reverse transcription (RT) was performed with 1 μg of RNA using ImProm-ll™ Reverse transcriptase (Promega, Madison, WI, United States). A duplicate set of PCR was performed using IP-Pro DNA Taq polymerase (Cosmogenetech, Seoul, Korea). The primers used for DEG and AS event validation, and the PCR product sizes are summarized in the Supplementary Table S2 and Supplementary Table S3 respectively.

### RBP Motif Enrichment Analysis

We sought to identify binding sites of splicing factors and other RBPs that were significantly enriched in differential exon skipping events. To assess the enrichment of RBPs near alternatively spliced exons, the differentially spliced exons from rMATS runs were compiled. These differentially spliced exons were scanned for RNA binding protein motifs using rMAPS2 (Hwang et al., 2020) with default parameters.

### Gene Ontology Analysis

We conducted two sets of GO analyses: the one with DEG results and the other with AS results. 1,479 Up-trend genes and 797 Down-trend genes from DEG analysis were compiled for the first set of GO analysis using PANTHER (Mi et al., 2021) with GO Ontology database release 2021–07–02 (The Gene Ontology, 2019). The host genes of significant skipped exon events from rMATS output were collected for the second set of GO analysis. For both sets of GO analysis, we examined BP (biological processes), MF (molecular functions), and CC (cellular components).

## Results

### Gene Expression Pattern Along the Tonotopic Axis of Cochlea Duct

To evaluate phenotypes associated with different regions along the longitudinal axis of the cochlea duct, we extracted mRNA from three different regions of the chick cochlea, base, middle and apex, and performed an RNA-seq experiment. This yielded 57 to 82 million read pairs per replicate, which were then aligned to the chicken genome. The transcript abundance was quantified, and FPKM values for each sample were obtained. Of 24,880 Ensembl-annotated genes, there were 13,045 (52.4%) in which at least one of the 9 samples had an FPKM ≥1. To assess position-specific gene expression that might be responsible for configuration and maintenance of the tonotopic axis of the cochlea duct, we identified differentially expressed genes on the three regions. We compared gene expression profiles from two different regions of cochlea at a time for all three regions. Since gene expression profiles originate from the same cochlea tissue despite being extracted from different regions, they should show intrinsically much similar gene expression patterns compared to those from different organs. Thus, we searched for DEGs with more relaxed criteria. We considered a 1.5 fold-change in expression level as a significant difference along three different regions.

**FIGURE 2:**
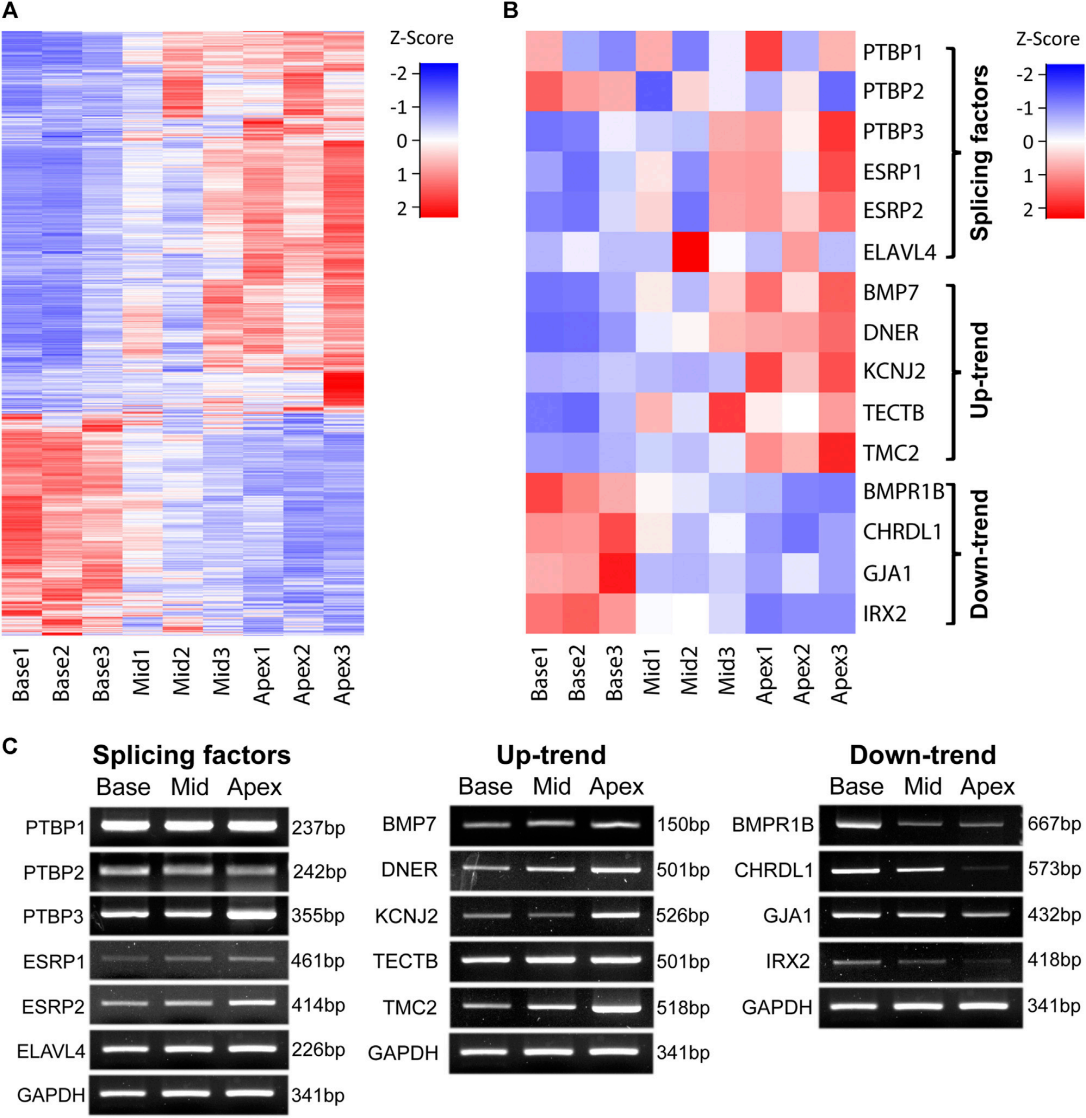
Expression patterns along the tonotopic axis. Gene expression patterns of genes along the tonotopic axis are shown **(A)** Expression patterns of all Up-trend and Down-trend genes. FPKM values of each gene are used for z-score normalization. **(B)** Expression patterns of validated genes in three different categories. FPKM values of each gene are used for z-score normalization. **(C)** RT-PCR images of the selected genes in each category. GAPDH is used as the internal control.

To identify genes that are responsible for tonotopic configuration along the cochlea duct, we searched for genes that gradually increase their expression by a factor of at least 1.5 from base to middle to apex regions and designated them as Up-trend. Likewise, we searched genes that gradually decrease their expression at least 1.5-fold from base to middle to apex regions and designated them as Down-trend. For these gene expression gradients, we identified 1,479 Up-trend genes and 797 Down-trend genes. The gene expression patterns along the regions of the cochlea are represented in Figure 2A which clearly shows up-trend (blue to red) and down-trend (red to blue).

Among those genes, we selected several genes that have been associated with cochlear development and hearing function for validation by RT-PCR. For Up-trend genes, we chose *BMP7*, *DNER*, *KCNJ2*, *TECTB*, and *TMC2* (Figure 2B). *BMP7* and *DNER* were shown to be expressed gradually higher towards the apex and regulate hair bundle morphology along the tonotopic axis in chicken basilar papilla (Kowalik and Hudspeth, 2011; Mann et al., 2014). *KCNJ2*, which encodes an inward rectifier potassium ion channel, is known to be a bona fide marker of apical hair cells in chicken cochlea (Son et al., 2015). *TECTB*, which is categorized as a supporting cell marker in chicken cochlea (Janesick et al., 2021), was shown to be expressed in a base-to-apex increasing gradient in mouse cochlea (Son et al., 2012; Yoshimura et al., 2014). *TMC2*, which encodes a component of the stereocilia mechanotransduction channel complex, was shown to be expressed higher at the apical regions of neonatal chicken and mouse cochlea (Kurima et al., 2015; Beurg et al., 2018; Janesick et al., 2021). For Down-trend genes, *BMPR1B*, *CHRDL1*, *GJA1*, and *IRX2* were selected. *BMPR1B*, encoding one of the receptors transducing BMP signaling, was shown to be expressed in the chicken hair cells (Lewis et al., 2018). *CHRDL1* was shown to be expressed higher at the base and promote basal hair bundle phenotypes in chicken basilar papilla (Frucht et al., 2011; Mann et al., 2014). Mutation in *GJA1* is shown to be associated with non-syndromic autosomal recessive deafness in human (Liu et al., 2001). *IRX2* was shown to be expressed higher in the base of chicken basilar papilla (Kowalik and Hudspeth, 2011; Janesick et al., 2021). We confirmed that their gene expression gradient pattern along the different regions of cochlea observed in RNA-Seq data agrees with the RT-PCR result as shown in Figures 2B,C. These results suggest that their differential expression along the cochlea duct is closely associated with the configuration and maintenance of the characteristic functions in tonotopy.

We next examined the expression patterns of AS factors including PTBPs, ELAVL, and ESPRs. RNA-seq data show that *PTBP3*, *ESRP1* and *ESRP2* are included in Up-trend genes and RT-PCR results confirmed their increasing expression gradient from base to apex (Figures 2B,C). Other AS factors including *PTBP1*, *PTBP2*, and *ELAVL4* were not included either Up- or Down-trend genes and did not show obvious expression gradient along the tonotopic axis by RT-PCR (Figures 2B,C). We reasoned that these AS factors take important roles in regulating gene expression in those regions in a sophisticated manner. In addition, considering subtle changes in controlling tonotopic differentiation along the longitudinal axis of the cochlea, we hypothesized that AS contributes to varying gene expression.

### Differential Alternative Splicing Is Common Along the Tonotopic Axis

**FIGURE 3:**
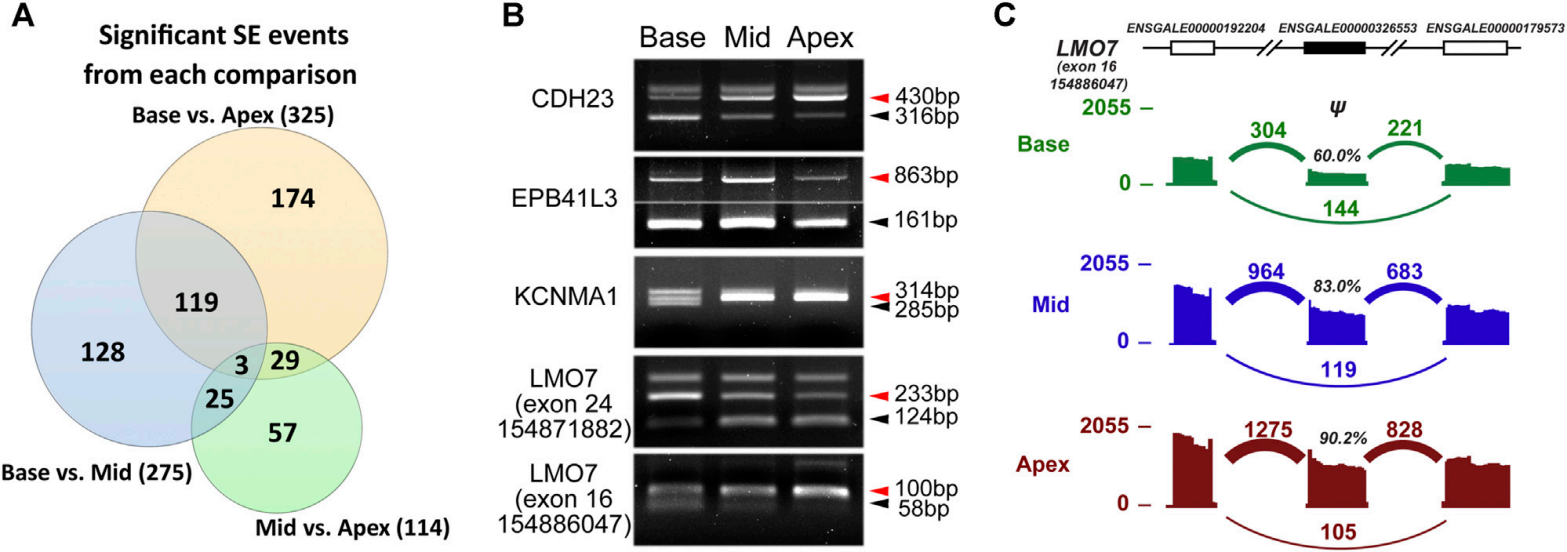
Comparison of AS profiles along the tonotopic axis. **(A)** Number of significant skipped exon (SE) events from each comparison at FDR <5% and |Δψ| ≥ 5%. **(B)** RT-PCR–based exon inclusion levels of five tonotopic signature exons. **(C)** A skipped exon event of the *LMO7* gene reported by rMATS (the last event in B) is shown with its flanking exons. Histograms represent exon read density and arcs represent splice junctions with the number of reads mapped to the junction indicated by the thickness of the arc. Both splice junction counts and exon read counts indicate that the target exon (solid) usage increases along the tonotopic axis.

We carried out differential AS analysis on paired-end RNA-Seq data from three regions of the chick cochlea. We used rMATS to identify five common types of AS events by comparing samples from two different regions, base vs middle, middle vs apex and base vs apex. Then, significant splicing events were identified at FDR <5% and |Δψ| ≥ 5% as described previously. The most common type of AS event was skipped exon events (SE), in which a cassette exon around two closely located exons is either included or skipped. From the three comparisons, we identified 275, 114, and 325 significant SE events, respectively (Figure 3A and Supplementary Table S4). Of these, 122 SE events were identified as significant on both base vs middle and base vs apex comparisons. Likewise, 32 SE events were significant on both base vs apex and middle vs apex comparisons and 28 SE events were significant on both base vs middle and middle vs apex comparisons.

We hypothesized the genes with AS events along the longitudinal axis of chick cochlea are related to tonotopic configuration and maintenance. Similar to Up-trend and Down-trend DEGs, some SE events took place along the axis of the cochlea in either an increasing or decreasing manner, from base to middle to apex, as summarized in Supplementary Table S4. We further verified these cochlear position-dependent SE events by RT-PCR for genes known to be crucial for hair cell development and function including *CDH23*, *EPB41L3*, *KCNMA1*, and *LMO7* (Figures 3B,C). *CDH23* is a component of the tip-link that is directly connected to the stereocilia mechanotransduction channel complex, mutations of which causes nonsyndromic (DFNB12) and syndromic (USH1D) hearing loss in humans (Di Palma et al., 2001). Interestingly, *CDH23* shows a base-to-apex increasing inclusion level and decreasing skipping level of the exon 32, suggesting that the protein region encoded by the exon 32 may provide the tonotopic properties of the tip-link crucial for frequency discrimination. *EPB41L3*, which was shown to be expressed in the tips of tallest stereocilia of mouse hair cells (Okumura et al., 2010), displayed higher inclusion levels in the middle of the cochlea compared to the base and apex (Figure 3B). *KCNMA1* encodes for the alpha subunit of the large conductance calcium-activated potassium channel (BK channel; also known as Slo), and its alternative splicing has been suggested to be important for frequency tuning along the tonotopic axis by influencing the channel gating properties (Navaratnam et al., 1997; Rosenblatt et al., 1997; Frucht et al., 2011). We also observed that *KCNMA1* showed differential exon usage along the tonotopic axis (Figure 3B). Our AS analysis also revealed that several different AS events can occur in the same gene. For example, *LMO7*, which was shown to be crucial for normal cuticular plate of hair cells and hearing function (Du et al., 2019), exhibited base-to-apex decreasing inclusion levels for exon 24 and the opposite base-to-apex increasing inclusion levels for exon 16 (Figures 3B,C).

In addition, we examined whether AS events are related to nonsense-mediated mRNA decay (NMD) pathway by introducing stop codons unexpectedly out of the original frame due to non-canonical AS events. As transcripts that have retained intron (RI) often contain premature stop codons, which can trigger the NMD (Yap et al., 2012), we searched differentially spliced RI events, then investigated the steady-state mRNA levels on different regions of the cochlea using our RNA-seq data. At FDR<=5%, we compiled 6 differentially spliced RI events and examined expression level changes of the host genes along the tonotopic axis. Genes that contain introns in the base region (5 genes with low expression level in the base region) showed an increase in their expression levels in the mid and apex regions while the gene that does not contain intron in the base region showed a decrease in its expression levels in the mid and apex region (Supplementary Figure S1). This pattern suggests that splicing change can regulate expression levels of genes by inducing the NMD pathway.

These results suggest that differential AS events along the cochlear duct act as a fundamental mechanism for establishing the tonotopy by altering morphological and functional properties of the proteins essential for frequency tuning. Although these genes represent gene expression gradients in AS events, they are not necessarily directly related to gene expression gradients at the transcription level.

### GO Enrichment Analysis on Genes Showing Differential Expression Levels and AS Events Along the Tonotopic Axis

**FIGURE 4:**
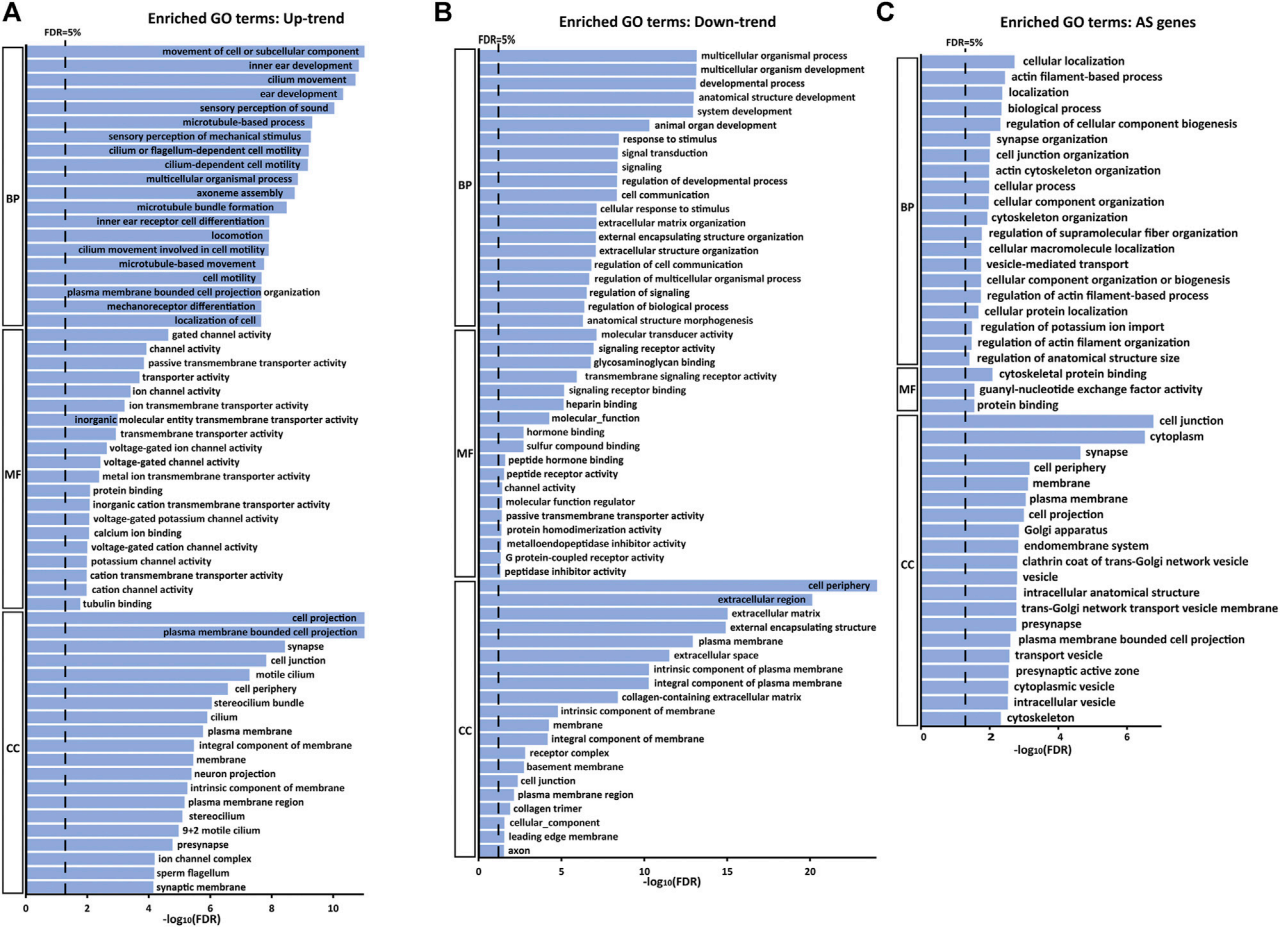
GO enrichment analysis of Up-trend genes, Down-trend genes, and differential exon skipping events. Up to top 20 significant GO terms (FDR <5%) in the GO biological processes (BP), molecular functions (MF) and cellular components (CC) categories are listed in the bar graph **(A)** Enriched GO terms with Up-trend genes. **(B)** Enriched GO terms with Down-trend genes. **(C)** Enriched GO terms with AS genes.

We further evaluated the characteristics of the genes detected from DEG analysis with Gene Ontology (GO) enrichment analysis using PANTHER (Mi et al., 2021). For the 1,479 Up-trend DEGs, enriched GO biological process terms are related to inner ear development, sound perception and microtubule (cilium)-related processes. Consistent with these terms, enriched molecular function terms include ion channel activity and transmembrane transporter activity and enriched cellular component terms are related to stereocilium and cilium. Meanwhile, for the 797 Down-trend DEGs, enriched GO biological process terms are related to the overall system developmental process, signal transduction, and extracellular structural organization. Similarly, enriched molecular function and cellular component terms include signaling receptor binding and extracellular component binding (Figures 4A,B). These results, which show that different GO terms are enriched between Up-trend and Down-trend DEGs, suggest that distinct groups of genes play more prominent roles in frequency tuning depending on the frequency ranges along the tonotopic axis. Genes with these enriched GO terms are listed in Supplementary Table S5.

We next performed GO enrichment analysis on genes with differential skipped exon events using PANTHER (Mi et al., 2021) (Figure 4C). Enriched GO biological process terms are mainly related to actin cytoskeleton organization, ion transport, and synapse organization. Consistent with these, enriched molecular function terms are related to cytoskeletal protein binding, and enriched cellular component terms are related to cell projection, the transport vesicle, and the synapse. These results suggest that differential AS events are essential for establishing the tonotopy by regulating the structural and functional characteristics of actin-based cytoskeletal structures such as stereocilia, ion channels essential for mechanoelectrical transduction and synaptic architectures transmitting the electrical signals to the brain. Genes with these enriched GO terms are listed in Supplementary Table S6.

### RNA Binding Protein Associated With Alternative Splicing Regulation Along With Tonotopic Axis

**FIGURE 5:**
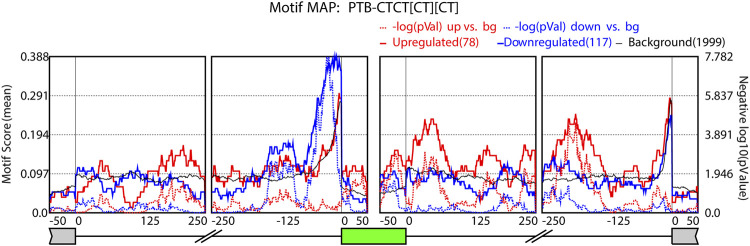
*PTBP1* RNA map. RNA map showing enrichment of PTB motif near alternatively spliced exons. The dotted lines indicate the significance of enrichment versus background in −log10(*p*-value). The number of events in each category (upregulated, downregulated, or background) is indicated in the parentheses.

To examine the relationship between expression gradients of AS events and regulatory RNA binding proteins (RBPs), we performed motif enrichment analysis using rMAPS2 (Hwang et al., 2020) with differential AS data generated from rMATS. We uploaded skipped exon events data from each comparison along the tonotopic axis to the rMAPS2 web server and generated RNA maps for over 200 RBPs. These RNA maps represent enrichment of motif sequence near the target exons of the SE events. Interestingly, PTB like (CT rich) binding motifs were enriched near alternatively spliced exons (Figure 5). PTB like binding motifs were enriched within the target exon and the downstream flanking intron regions when the inclusion of the target exon is promoted (solid red line shows peaks with small *p*-value) while the PTB like motifs were enriched in the upstream flanking intron regions when the inclusion of the target exon is suppressed (solid blue line shows peaks with small *p*-values). We also examined the enrichment of ESRP 1/2 motifs (Supplementary Figure S2). There was a strong enrichment for these ESRP motifs (solid red line) in the upstream intron of upregulated exons. The differential expression of the *ESRP 1/2* and *PTBP3* together with the enrichment of their binding motifs and their positional enrichment relative to the regulated exons further suggest that the tonotopic related AS may be mediated by these splicing factors.

## Discussion

We report that alternatively spliced transcript variants encoding different protein isoforms are closely related to the configuration along the tonotopic axis of the chicken cochlea as well as differentially expressed genes. Unlike DEGs on two different organs or two different treatments, DEGs on the different regions along the longitudinal axis of the cochlea show subtle changes on the expression level. By applying relaxed criteria, we found DEGs that represent gene expression gradients along the tonotopic axis, from base to middle to apex regions where sounds are detected from higher frequencies to lower frequencies. We performed RNA-Seq analysis and identified 1,479 Up-trend and 797 Down-trend genes that might be responsible for configuring the tonotopic axis and maintaining the cochlea’s function in detecting different frequencies of sound. Among DEGs, as expected, we identified genes that were responsible for regulating membrane proteins and ion channels that affect structural formation, signaling, and sensory function. We further validated our RNA-seq analysis results with RT-PCR for some of DEGs to confirm that the RNA-seq data set reflected actual gene expression at the transcription level.

GO analysis using Up-trend and Down-trend DEGs shows that different GO terms are enriched in each group. In Up-trend DEGs expressed at higher levels in the apex, GO terms related to the “inner ear” are enriched. For example, GO terms among enriched biological processes include inner ear development, ear development, sensory perception of sound, and inner ear receptor cell development. In addition, enriched GO terms in molecular function and cellular component are mostly related to hair cell function and morphology such as ion channel activity and stereocilium. Consistent with these GO analysis results, Up-trend DEGs include many genes related hearing loss such as *MYO7A*, *CLRN1*, *WHRN*, *FSCN2*, *PCDH15*, *PLS1*, *CIB2*, *USH1C*, *THRB*, and *PTPRQ*. On the other hand, in Down-trend DEGs expressed higher levels at the base, enriched GO terms include broad developmental and biological processes such as system development, multicellular organismal processes, and signal transduction. GO terms enriched in molecular function and cellular component are also general biological terms but not specific to the inner ear. These results suggest that tonotopy-specific features are mainly dictated by Up-trend DEGs, while general developmental and biological processes are maintained by Down-trend DEGs.

We recognized that there are a few differentially expressed splicing factors along the tonotopic axis and we sought to identify how AS influences in expressing different phenotypes along the cochlea duct. Using paired-end RNA-Seq data from three different regions of chick cochlea, we identified AS events, focusing on the most common type of AS events, SE. We confirmed some previously known AS events along the tonotopic axis, such as of the *KCNMA1* gene, which was reported to have 7 different splicing sites (Navaratnam et al., 1997; Rosenblatt et al., 1997; Miranda-Rottmann et al., 2010). We validated the exon inclusion event at the last splicing site, site 7, by RT-PCR (Figure 3B). Furthermore, our AS analysis showed three additional AS events in the *KCNMA1* gene. Two of them show low inclusion levels throughout the tonotopic axis, while the other shows high inclusion levels at the base and apex compared to the middle cochlear region. This suggests that different splicing variants generate distinct BK channels that influence frequency tuning along the tonotopic axis. In addition to *KCNMA1*, many genes showed differential AS along the tonotopic axis. Notably, *CDH23*, encoding a component of the tip-link, has been known to have an inner ear-specific exon included only in the hair cells in mouse (Wang et al., 2016). Among 69 exons in the mouse *Cdh23* gene, exon 68 is subjected to AS, so that two *Cdh23* splicing variants, *Cdh23* (+68) and *Cdh23* (−68), are produced in the mouse hair cells. Our analysis show that this inner ear-specific AS events of *CDH23* gene also occur in chicken. The chick *CDH23* gene has 33 exons, and we found *CDH23* (+32) and *CDH23* (−32) splicing variants in chick basilar papilla. Interestingly, the amino acid sequences of the inclusion exon (exon 68 in mouse and exon 32 in chicken) show 96.4% similarity, suggesting that the inner ear-specific exon inclusion is conserved in avians and mammals. Moreover, our AS analysis further revealed that this AS event occur differentially along the tonotopic axis (Figure 3), suggesting that this differential AS events on *CDH23* gene determines the conformational properties of the tip-link essential for the fine-tuned frequency discrimination along the tonotopic axis. In addition to inner ear specific genes, we found that *HNRNPH1*, encoding the heterogeneous nuclear ribonucleoprotein H, shows a tonotopically graded splicing event, with higher inclusion levels at the middle and apex, suggesting that HNRNPH1 influences pre-mRNA processing differentially along the tonotopic axis. Binding motif sequences of the HNRNPH1, for example, are enriched in the upstream intron and the downstream intron of the upregulated exons and in the upstream intron of downregulated exons, near alternatively spliced exons (Supplementary Figure S3).

When we performed GO analysis with the genes showing significant SE events, GO terms related to actin cytoskeleton organization were enriched, including genes such as *EPB41L3*, *EPB41L1*, and *LMO7*. EPB41L3 is shown to be expressed at the tips of tallest stereocilia in the mouse hair cells (Okumura et al., 2010). This localization is similar with those of WHRN and MYO15, whose mutations cause hearing loss in humans and mice. Interestingly, the stereociliary localization of EPB41L3 is abolished in *Whrn* and *Myo15* mouse mutants (Okumura et al., 2010). These results suggest that these proteins may interact at the tips of tallest stereocilia, and expressing different *EPB41L3* isoforms along the tonotopic axis may alter physical properties of the stereocilia by affecting the protein-protein interactions. LMO7 is expressed in the cuticular plate, the actin-rich apical surface of hair cell body, in the mouse cochlea, and *Lmo7* Knockout mice suffer from hearing loss due to actin cytoskeletal defects in the cuticular plates and stereocilia (Du et al., 2019). We found two different AS events exhibiting opposite trends along the tonotopic axis, suggesting that unique combinations of at least four different *LMO7* isoforms are present along the cochlear duct, which may regulate the actin cytoskeletal properties along the tonotopic axis. We confirmed that SE events were commonly observed in different regions of chick cochlea and that they were an important gene regulatory mechanism that supports variation along the tonotopic axis. Nevertheless, we noticed that gene expression gradients along the tonotopic axis were not necessarily correlated between DEGs and AS of a gene.

We further identified regulatory RBPs, which may play essential roles in differential AS events along the tonotopic axis. We noted that PTB motif sequences are enriched around skipped exons in either the upstream intron region of the exon or in the exon and downstream intron regions. While functional validation of PTBP1 in the AS events along the cochlea warrants further investigation, it is noteworthy that the inclusion of exon 68 of *Cdh23* gene, which is specific to the cochlear hair cells, is regulated by PTBP1 (Wang et al., 2016; Li et al., 2020). In addition, *PTBP1* expression in the spiral (cochlear) ganglion neurons suggests its possible role in the regulation of neuronal gene expression and differentiation in the developing mouse cochlea (Polydorides et al., 2000). Furthermore, it has been shown that a mutation in *Srrm4*, which encodes a splicing factor, causes hearing loss in mouse (Nakano et al., 2012). Since the binding sites for PTBP1, pyrimidine-rich motifs, are often present in introns that flank SRRM4-regulated exons and PTBP1 inhibits the SRRM4-dependent exon inclusion, it is possible that PTBP1 regulates AS events in the cochlea by competing with SRRM4 for the pyrimidine-rich motifs. Together, our RBP analysis implies functional significance of PTBP1 in the differential AS events along the tonotopic axis of the cochlea. (Frucht et al., 2011)

We also found that the intensity of transcriptional expression of splicing factors does not necessarily correspond to the gene expression levels along the tonotopic axis, suggesting that there could be additional regulatory factors involved in AS such as signaling genes and ligand-binding proteins (Kim et al., 2005; Ushiyama et al., 2010; Jules et al., 2013).

Given the lists of genes from DEG and AS analyses, we identified biological processes that are represented with those genes and opened the possibility to find the functions of some genes whose roles are not yet fully understood in establishing the tonotopy. Since many morphological and functional features are conserved in avian and mammalian cochlea, DEGs and AS events identified in the chicken cochlea will greatly help exploring the mechanisms contributing to cochlear development and function as well as hearing loss in mammals such as mice and humans. Moreover, comparative analysis of DEGs and AS events between chicken and mouse cochlea may shed light on uncovering the mechanism of hair cell regeneration occurred specifically in chicken. In summary, the overall gene expression profiles, specifically AS events along the tonotopic axis of the chick cochlea, are orchestrated by complex gene expression mechanisms in addition to the differential expression of genes depending on different regions of the cochlea. We show that the detection of subtle frequency differences of sound throughout the longitudinal axis of the chicken cochlea is sophisticatedly regulated by differential expression of genes and that AS takes an important role in regulating gene expression gradients along the tonotopic axis.

## Data Availability

The datasets presented in this study can be found in online repositories. The names of the repository/repositories and accession number(s) can be found below: Gene Expression Omnibus (https://www.ncbi.nlm.nih.gov/geo/), accession number GSE176521.
