# Supplementary material for: Position Specific Alternative Splicing and Gene Expression Profiles Along the Tonotopic Axis of Chick Cochlea
Source: Front Mol Biosci. 2021 Sep 8;8:726976. doi: 10.3389/fmolb.2021.726976 (PMC8456117; doi:10.3389/fmolb.2021.726976)
Supplement: Supplementary file 2 [file Table2.DOCX]

**Supplementary table 2** Primers for DEG genes

| **Gene name** | **Primer (F-R)** | **PCR product size (bp)** |
| --- | --- | --- |
| BMP7 | AAAACAAGCAGCCCTTCACG | 150 |
|  | GGTGTCAAACATCGCAGAGA |  |
| BMPR1B | CAGATGGAGCAGCGATGAGT | 667 |
|  | CCACTGTAGCTTCTCCGAGG |  |
| CHRDL1 | CGCGGAGTTCTACCAGATCC | 573 |
|  | GATCTGAGCTTCTCCCTCGC |  |
| DNER | TTGATGGCAGAAGCGTCACT | 501 |
|  | CTGGGCACTGACAGGTGAAT |  |
| ELAVL4 | GGTGGACTGCGCAATAGGTA | 226 |
|  | TCGGAAGCAAAGAGAGCGTT |  |
| ESPR1 | ATCCTCTTTGTGACCACGGC | 461 |
|  | TCTGTCATGGCCGGAACATC |  |
| ESRP2 | GTTGTGGATCTCCCCAGCAA | 414 |
|  | CTCCTCCGCTTCATCTGTCC |  |
| PTBP1 | TCGTTCCTGTTATTCCGGCG | 237 |
|  | TGACGTCACTGGGGAGCTTA |  |
| PTBP2 | GAACACCACCCACCCTTCAC | 242 |
|  | CGGAGAGCCTGCGAGAAAAT |  |
| PTBP3 | GCTCTGATCGCCTTCTGCTC | 355 |
|  | TGACATCTGCTTCAGTGGCAT |  |
| GAPDH | GACAACTTTGGCATTGTGGA | 341 |
|  | GCATCCTAGGATACACAGAGGA |  |
| GJA1 | ATGGGTTTAGCCTGAGTGCC | 432 |
|  | GCCCCATCCTATTCTGCTCC |  |
| IRX2 | TCAAGAGCCAGAACTTGGGC | 418 |
|  | CCTACTTGCATTGCTGCGTG |  |
| KCNJ2  (Son et al, 2015, PNAS) | AAGCCACTGCCATGACTACC | 527 |
|  | TGAAGGCAAGCAGACCTGAG |  |
| TECTB  (Janesick et al, 2021, Cell report) | TCCCAGACCTGTCACCTAAA | 488 |
|  | CTTCTCCATTGTCGTCCATGAG |  |
| TMC2  (Janesick et al, 2021, Cell report) | ACCAGGTCTCGTGGGTATAA | 518 |
|  | GATTTACTTGCACCGAGTACGA |  |
