# Supplementary material for: Position Specific Alternative Splicing and Gene Expression Profiles Along the Tonotopic Axis of Chick Cochlea
Source: Front Mol Biosci. 2021 Sep 8;8:726976. doi: 10.3389/fmolb.2021.726976 (PMC8456117; doi:10.3389/fmolb.2021.726976)
Supplement: Supplementary file 4 [file Table3.DOCX]

**Supplementary Table 3** Primers for AS validation

| **Gene name** | **Primer (F-R)** | **Target exon size (bp)** | **PCR product size (bp)** |
| --- | --- | --- | --- |
| CDH23 | GCTGCCAAACCTGAGGATGA | 114 | 430 (inclusion) |
|  | CGCAGTCGGCAGTTAGAGTC |  | 316 (skipped) |
| EPB41L3 | CAGGAAGGAGAATCTGCTGACA | 702 | 863 (inclusion) |
|  | AACTCAGTTGGCTTTCTGGC |  | 161 (skipped) |
| KCNMA1 | GTTTGAGCTTGTGCCGACAG | 29 | 314 (inclusion) |
|  | TCTGATTGGCCATGTGGGTG |  | 285 (skipped) |
| LMO7 (154871882) | TCTCAACCAGGGTCGCAGAT | 109 | 233 (inclusion) |
|  | CTGAGATAGCAGTCGTTGCAG |  | 124 (skipped) |
| LMO7 (154886047) | TGGCATGCAAGGAGATCTTGG | 42 | 100 (inclusion) |
|  | CATTGCAGGCTCTGGACCTC |  | 58 (skipped) |
