# Supplementary material for: Position Specific Alternative Splicing and Gene Expression Profiles Along the Tonotopic Axis of Chick Cochlea
Source: Front Mol Biosci. 2021 Sep 8;8:726976. doi: 10.3389/fmolb.2021.726976 (PMC8456117; doi:10.3389/fmolb.2021.726976)
Supplement: Supplementary file 7 [file DataSheet1.DOCX]

Position Specific Alternative Splicing and Gene Expression Profiles along the Tonotopic Axis of Chick Cochlea

Supplementary Material


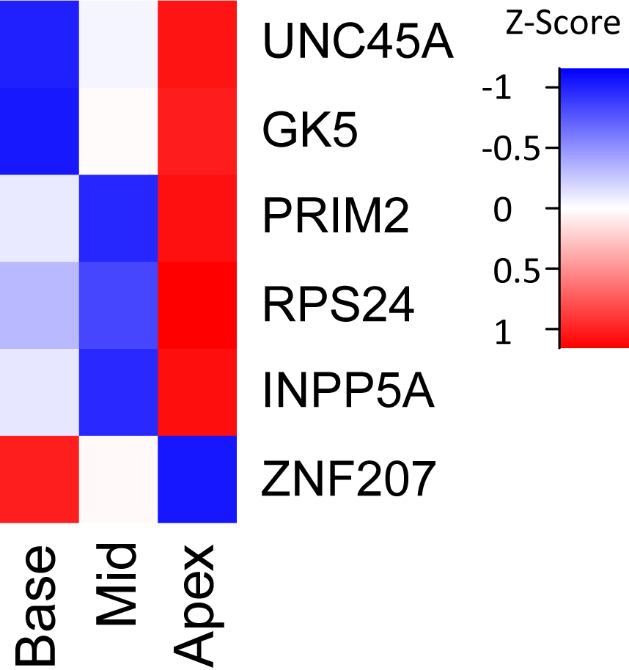


**Supplementary Figure 1** Heatmap comparison of the mRNA levels of genes whose retained intron (RI) events are differentially spliced in different regions. First five genes have higher intron retention rate in the base region than mid or apex region. The last gene has lower intron retention rate in the base region.


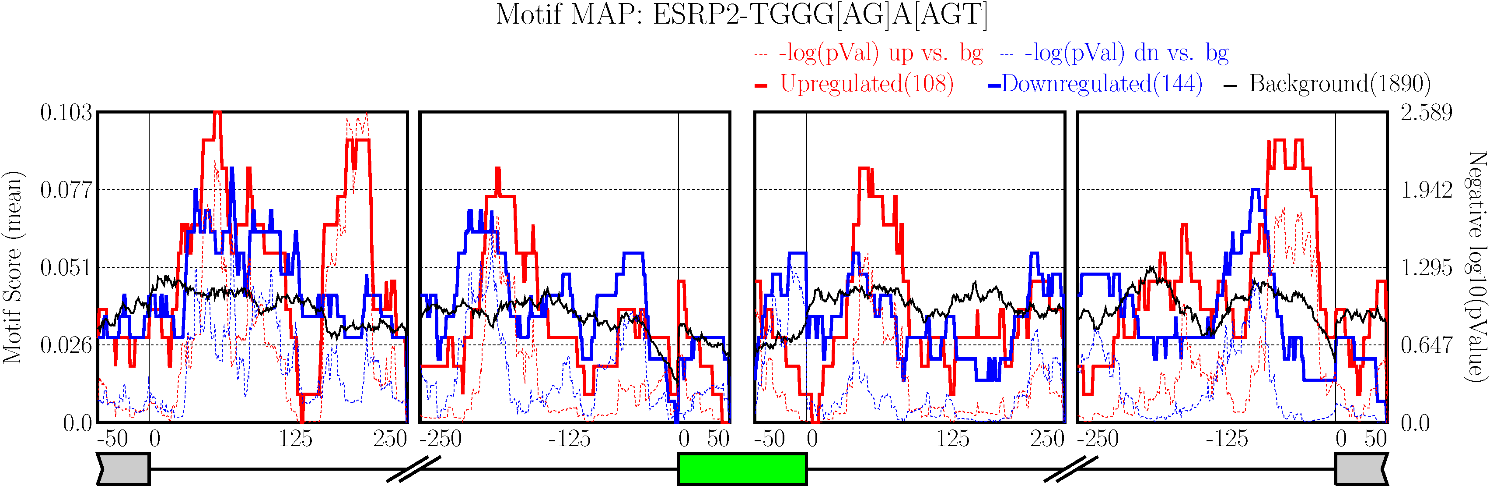

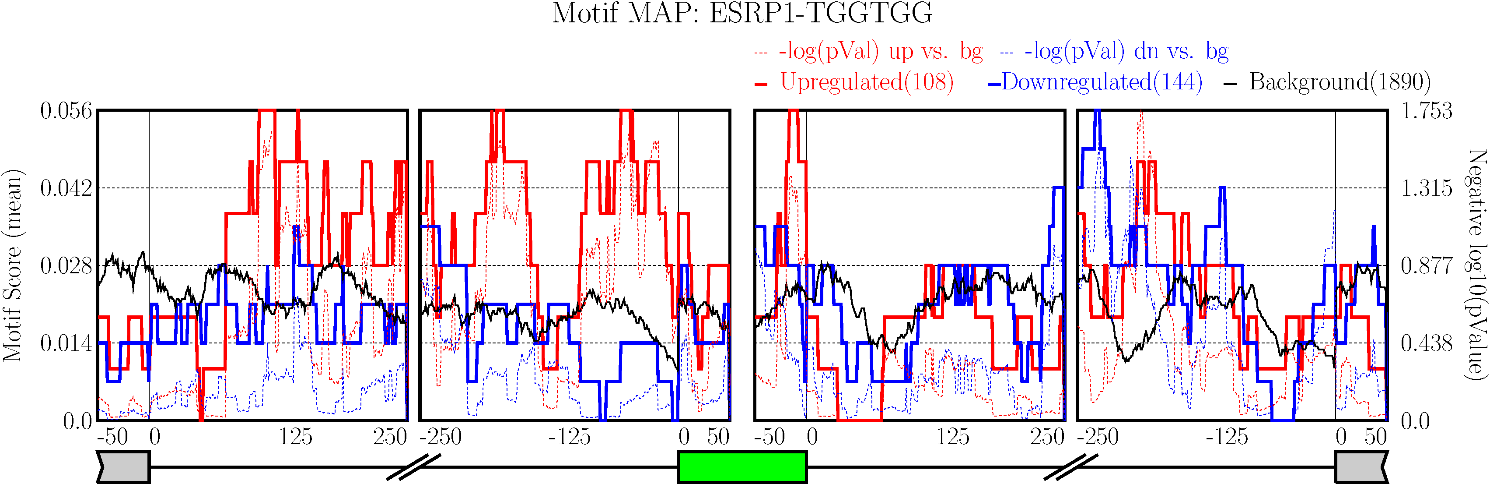


**(B)**

**(A)**

**Supplementary Figure 2** RNA maps for splicing factors. **(A)** RNA map showing enrichment of ESRP1 motif near alternatively spliced exons. ESRP1 binding motifs are enriched in the upstream intron and the body of the upregulated exons. **(B)** RNA map showing enrichment of ESRP2 motif near alternatively spliced exons. ESRP2 binding motifs are enriched in the upstream intron of the upregulated exons.

The dotted lines indicate the significance of enrichment versus background in −log10(P-value). The number of events in each category (upregulated, downregulated, or background) is indicated in the parentheses.


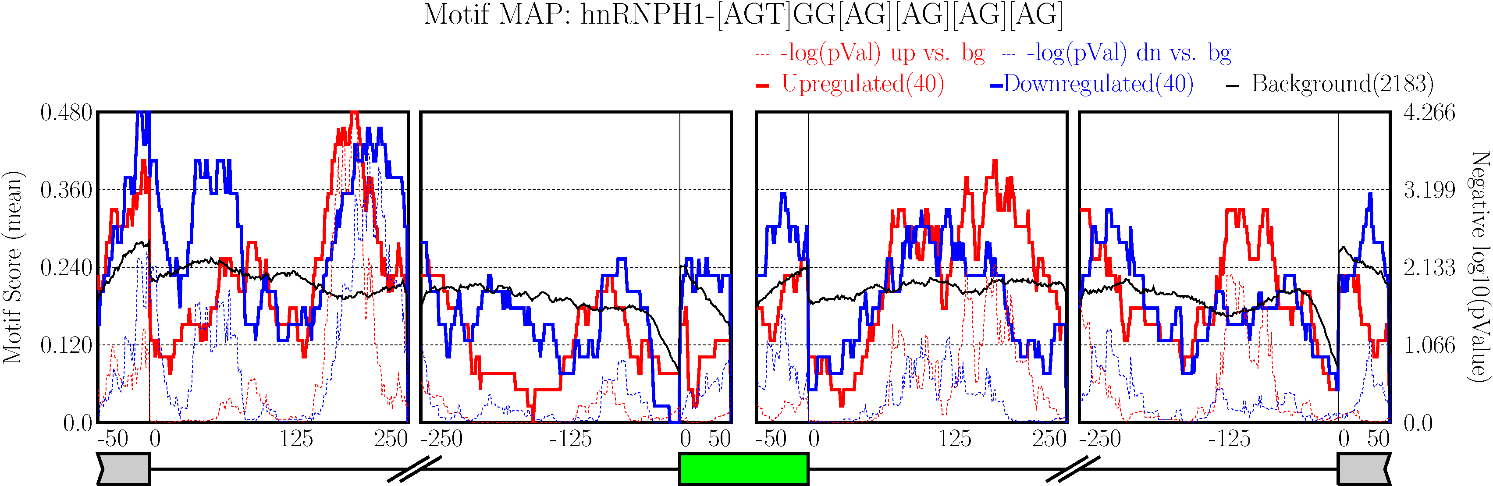


**Supplementary Figure 3** RNA map showing enrichment of hnRNP H1 motif near alternatively spliced exons. hnRNP H1 binding motifs are enriched in the upstream intron and the downstream intron of the upregulated exons (red lines) and in the upstream intron of downregulated exons (blue lines). The dotted lines indicate the significance of enrichment versus background in −log10(P-value). The number of events in each category (upregulated, downregulated, or background) is indicated in the parentheses.
